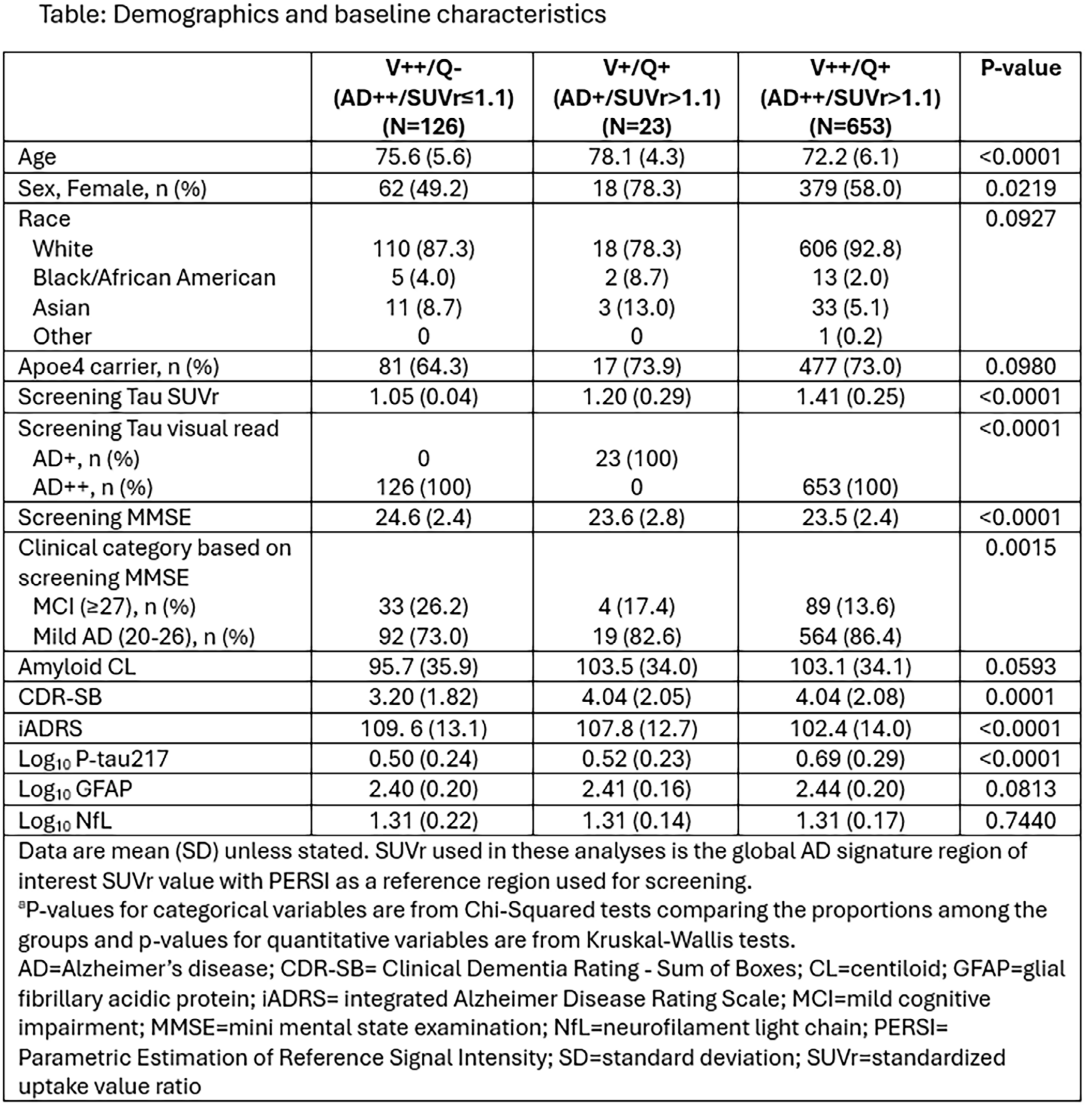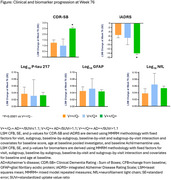# Characterizing early symptomatic AD patients by visual and quantitative tau profiles

**DOI:** 10.1002/alz70856_106941

**Published:** 2026-01-11

**Authors:** Amanda Morris, Min Jung Kim, Michael Pontecorvo, Sergey Shcherbinin, Leonardo Iaccarino, Samantha C. Burnham, John R. Sims, Dawn A. Brooks, Emily C. Collins, Mark A. Mintun, Ming Lu

**Affiliations:** ^1^ Eli Lilly and Company, Indianapolis, IN, USA

## Abstract

**Background:**

In the TRAILBLAZER‐ALZ 2 study (NCT04437511), tau pathology at screening was evaluated with flortaucipir Positron Emission Tomography (PET) with a combination of visual read (V) and quantitative (Q) standardized uptake value ratio (SUVr) approaches. In most cases, participants had an advanced Alzheimer's disease (AD) tau visual pattern (AD++) with an AD‐signature weighted neocortical SUVr>1.1 (V++/Q+). A minority of participants presented other V/Q profiles, namely an AD++ visual read with an SUVr**≤**1.1 (V++/Q‐) and a moderate AD tau pattern (AD+) with an SUVr>1.1 (V+/Q+). The objective of these analyses was to characterise these populations.

**Method:**

All available TRAILBLAZER‐ALZ 2 placebo‐treated early symptomatic AD participants were included. Baseline characteristics and progression on both clinical scales (Clinical Dementia Rating ‐ Sum of Boxes [CDR‐SB], integrated Alzheimer Disease Rating Scale [iADRS]) and biomarkers (*p*‐tau217, glial fibrillary acidic protein [GFAP], neurofilament light chain [NfL]) at Week 76 (using mixed model repeated measures methodology), of the V++/Q‐ and V+/Q+ groups were compared to each other and to the rest of the study population (V++/Q+).

**Result:**

The V++/Q‐ group comprised 15.7% (126/802) and the V+/Q+ group 2.9% (23/802) of the placebo‐treated study population with characteristics as shown in the Table. At Week 76, all groups were at risk of clinical progression, although changes from baseline in CDR‐SB and iADRS for the V++/Q‐ and V+/Q+ groups were less compared to the rest of the population (significantly different between the V++/Q‐ and V++/Q+ groups; *p* <0.0001 for both CDR‐SB and iADRS) (Figure). Progression for both AD‐specific and non‐AD specific biomarker progression was similar across all groups.

**Conclusion:**

The observation of V++/Q‐ and V+/Q+ participants being older, milder in AD pathology while at a similar clinical stage at screening compared to V++/Q+ participants may suggest the effect of age‐related comorbidities. Participants in the V++/Q‐ and the V+/Q+ groups showed clinical/pathological progression, although not at the level of those in the V++/Q+ group.